# Cryo-electron tomography and 3-D analysis of the intact flagellum in *Trypanosoma brucei*

**DOI:** 10.1016/j.jsb.2012.01.009

**Published:** 2012-05

**Authors:** Johanna L. Höög, Cédric Bouchet-Marquis, J. Richard McIntosh, Andreas Hoenger, Keith Gull

**Affiliations:** a The Boulder Laboratory for 3-D Electron Microscopy of Cells, MCD-Biology, University of Colorado at Boulder, Boulder, CO 80309-0347, USA; b The Sir William Dunn School of Pathology, University of Oxford, South Parks Road, Oxford OX1 3RE, UK

**Keywords:** Flagellum, *Trypanosoma brucei*, Kinetoplastid, Paraflagellar rod, Cryo-electron tomography, Frozen hydrated sections

## Abstract

*Trypanosoma brucei* is a uni-cellular protist that causes African sleeping sickness. These parasites have a flagellum that is attached to the cell body and is indispensible for its motility. The flagellum consists of a canonical 9 + 2 axoneme and a paraflagellar rod (PFR), an intricate tripartite, fibrous structure that is connected to the axoneme. In this paper we describe results from cryo-electron tomography of unperturbed flagella. This method revealed novel structures that are likely involved in attaching the flagellum to the cell. We also show the first cryo-electron tomographic images of a basal body in situ, revealing electron dense structures inside its triplet microtubules. Sub-tomogram averaging of the PFR revealed that its distal region is organized as an orthorhombic crystal.

## 1 Introduction

*Trypanosoma brucei* (*T. brucei*) is a single cell parasite that causes the African sleeping sickness (human African trypanosomiasis), endemic in sub-Saharan Africa. *T. brucei* is transmitted by the tsetse fly (*Glossina* spp.). Throughout 2009, an estimated 30.000 humans were infected (WHO, 2010). Currently, available treatments have severe side effects, and resistance against these drugs is increasing (Pyana et al., 2011; Wilkinson and Kelly, 2009). Detailed structural and cell biological studies of these parasites might reveal important novel drug targets. Furthermore, *T. brucei* belongs to the kinetoplastids, a group of protozoa that is evolutionary distant from humans, which renders it an excellent organism to study cellular evolution in eukaryotes.

**Fig.1:**
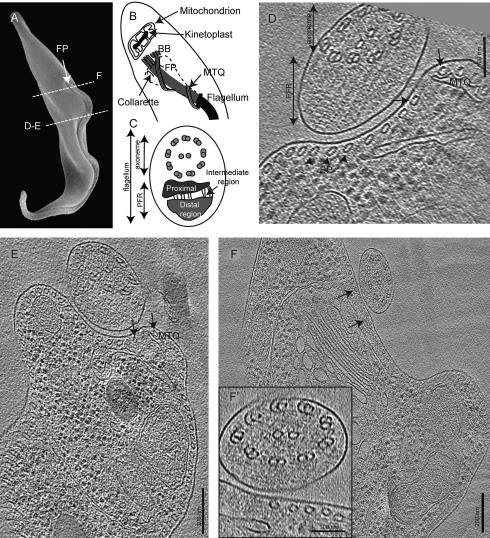
Vitreous sections of the flagellum in *Trypanosoma brucei.* (A) A scanning electron micrograph shows *T. brucei*’s elongated cell shape and attached flagellum that grows out of the flagella pocket (FP) (with permission from Lacomble et al. (2009)). (B) A cross-sectional cartoon of the cell’s posterior end, with the FP around which the microtubule quartet (MTQ) wraps. At the proximal end of the flagellum, the basal bodies (BB) with the associated kinetoplast-containing mitochondrion. At the base of the flagellum inside the FP is the filamentous collarette structure. (C) The flagellum consists of the axoneme and the paraflagellar rod (PFR). (D–F) Slices from cryo-tomograms of frozen hydrated sections, showing the axoneme, PFR and sub-pellicular MTs (arrowheads; SP). Note the distance between flagellar and cellular membranes. (F) The PFR in this slice is thinner than in others, suggesting that this section was cut where the flagellum emerged from the flagellar pocket, the site of the PFR proximal end. This interpretation is supported both by the large gap in the sub-pellicular microtubules (arrows) underlying the membrane and the presence of the Golgi apparatus, which are also found in this region. (F′) zoomed in image of the flagellum of panel F. Thickness of slice in Z: (C) 50 nm (D) 1 nm and (E) 30 nm.

*T. brucei* cells maintain their characteristic slender shape due to the sub-pellicular microtubule array, an arrangement of mostly parallel microtubules located directly under the cell membrane (Gull, 2003; Robinson et al., 1995). The cells are motile due to a single flagellum that grows from a basal body situated below a secretory organelle called the flagellar pocket, which is located near the cell’s posterior (Fig. 1A) (Lacomble et al., 2009; Webster, 1989). The flagellum is attached to the cell body over its entire length, except for a short segment that protrudes beyond the cell’s anterior end. This attachment is important because silencing of the *fla1* gene by RNAi, which detaches the flagellum, decreases cellular viability and causes failure in cytokinesis (LaCount et al., 2002; Nozaki et al., 1996). Attachment is accomplished by a “flagellum attachment zone” (FAZ), a specialization of the cell’s cortex positioned on the inside of the plasma membrane just opposite the flagellum. This zone includes a gap in the sub-pellicular microtubule array that contains the so-called “microtubule quartet” (MTQ), a sub-set of the subpellicular MTs that are nucleated at the base of the flagellar pocket, and are anti-parallel to the other MTs (Fig. 1B) (Sherwin and Gull, 1989; Webster, 1989). This gap also contains the “FAZ filament”, an electron dense fiber that runs parallel to the MTQ, and the macula adherens, junctional complexes between cell body and the flagellum (Vickerman, 1969). Nonetheless, the ways in which these cytoplasmic specializations might bind the flagellum to the cell surface have remained obscure. The images obtained in this study reveal novel structures that may be important for flagellar attachment.

The flagellum in *T. brucei* comprises two major structures, the conserved 9 + 2 axoneme (9 doublet microtubules and two central pair microtubules) and the more kinetoplastid specific paraflagellar rod (PFR; (Vaughan, 2010) Fig. 1C). The PFR is a paracrystalline fiber that is arranged parallel to the axoneme along the extra-cellular part of the flagellum (de Souza and Souto-Padron, 1980; Portman and Gull, 2010; Vickerman, 1962). The PFR constitutes a platform for metabolic enzymes and signaling factors (Oberholzer et al., 2007; Pullen et al., 2004; Ridgley et al., 2000). It is essential for motility (Bastin et al., 1998; Santrich et al., 1997) and thus, cell viability in the bloodstream form that causes the human infection (Broadhead et al., 2006; Griffiths et al., 2007).

Previous structural studies on the PFR have revealed three structurally distinct regions: the proximal, intermediate and distal domains (Farina et al., 1986). They also showed that the PFR has links to axonemal doublet microtubules numbered 4–7 and to the flagellar attachment zone (de Souza and Souto-Padron, 1980; Koyfman et al., 2011; Sherwin and Gull, 1989). The PFR distal region consists of periodically arranged crossing filaments (de Souza and Souto-Padron, 1980; Rocha et al., 2010; Sant’Anna et al., 2005), that change their angles relative to one another, depending on the extent of flagellar bending in that region (Koyfman et al., 2011; Rocha et al., 2010).

Here we present the first structural 3D analysis of the intact *T. brucei* flagellum by cryo-electron tomography (cryo-ET). We have investigated both vitreous sections and whole, plunge-frozen cells, each of which provides structural preservation that is the best currently possible (Al-Amoudi et al., 2004; Hoenger and Bouchet-Marquis, 2011; Leis et al., 2009). Studying cells in this near-to-native state has provided insight at the macromolecular level into the flagellum attachment and the structure of the PFR. The 3D volumes obtained from cryo-ET were further analyzed by averaging sub-tomogram volumes from repetitive and structurally identical areas. The resulting increase in signal-to-noise ratio has helped to identify repeating structures in the distal region of the PFR.

## 2 Material and methods

### 2.1 Cell culture and sample preparation

*T. brucei brucei*, procyclic form (Lister 429), were maintained in logarithmic growth, using SDM79 medium with 20% FCS at 28 °C, as in (Höög et al., 2010). To prepare cells for whole cell cryo-ET, 4 μl of cells at a density of ∼3 × 10^6^ cells/ml were pipetted onto a glow-discharged holy carbon grid (4 × 4 μm holes; Quantifoil, Jena, Germany) and mixed with 1 μl of concentrated 10 nm colloidal gold particles (Ted Pella, Redding, CA, USA), blotted and plunged into liquid ethane.

For the preparation of vitreous sections, cells were concentrated by centrifugation for 2 min at 600*g*, and then resuspended in 20% dextran and 0.1% sucrose in medium. After 30 min in this mixture, a majority of cells were still swimming. We cryo-immobilized the cells by high pressure freezing (BalTec HPM-010) within 2–3 min after resuspending the pellets, thus we can assume that the cells were healthy at the time of freezing.

#### 2.1.1 Cryo-imaging of plunge-frozen whole cells

The physical forces encountered during blotting of excess liquid from the grids immediately before freezing destroyed the vast majority of cells. Therefore, a selective search for *T. brucei* cells of normal cell shape was performed. Cryo-micrographs and –tomograms were recorded on intact cells that were situated so that the thin, anterior end and flagellum lay within one of the 4 μm holes in the carbon film. Cryo-electron microscopy (cryo-EM) was performed on a Tecnai F30 electron microscope (FEI Company Ltd., Eindhoven, The Netherlands) operated at 300 kV. Tomographic tilt series were collected between ±60° in 1.5° increments, at a defocus of −4 or −6 μm, using the data acquisition software SerialEM (Mastronarde, 2005). Tilt-series were recorded at a magnification of 27,500× using a GATAN UltraCam, lens-coupled, 4 K CCD camera attached to a Tridiem Gatan Image Filter (GIF: operated at zero-loss mode with an energy window of 20 eV; Gatan Inc., Pleasanton, CA, USA). The original images were binned by 2, resulting in micrographs of 2048 × 2048 pixels with a pixel size corresponding to 0.76 nm on the specimen. The maximum total dose used for a tilt-series was 90 e/Å^2^, corresponding to about 1–2 e/Å^2^ for each micrograph. Tilt-series were corrected for contrast-transfer function (CTF) modulations (Xiong et al., 2009) and tomograms were reconstructed using IMOD (Kremer et al., 1996).

#### 2.1.2 Cryo-sectioning and imaging of cryo-sections

Dome-shaped carriers containing vitrified *T. brucei* obtained by high pressure freezing were mounted in an FCS cryo-chamber of an ultracut UCT microtome (Leica, Vienna, Austria) using a chuck of our own construction. This chuck holds the carrier so that the protruding domeshape of vitreous material can be trimmed and sectioned, and avoids removing the specimen from its carrier to mount it on a pin using cryo-glue (Richter, 1994). The specimens were trimmed into a pyramid shape in order to obtain a square surface (100 μm side) using a 45° cryo-trimming knife (Diatome, Bienne, Switzerland). Then, 50 nm (nominal feed) thin sections were produced using a 45° cryo-diamond knife (Diatome) with a clearance angle of 6°. The cutting was performed at −155 °C with a cutting speed of 0.6 mm/s (Bouchet-Marquis and Hoenger, 2011). During cutting, an anti-static device (Diatome) was used to enhance the sliding of the sections on the surface of the knife. The sections were collected on 200-mesh carbon-coated grids and squeezed between two cooled polished metal surfaces in order to attach them to the carbon film. When possible, the grids were observed in the electron microscope directly after sectioning to limit ice crystal contamination during storage.

The vitrified sections were transferred at liquid nitrogen temperature into a Gatan-626 or 914 cryo-holder (Gatan Inc., Warrendale, PA, USA), which maintains a temperature of approx. −180 °C to preserve the vitrified state. The proper vitrification of the specimen was tested by electron diffraction. Cryo-EM and cryo-ET on vitrified sections was performed on a Tecnai F20 electron microscope (FEI Company Ltd., Eindhoven, The Netherlands) operated at 200 kV. Data were recorded with an UltraScan 4000, 4Kx4K Gatan CCD camera (Gatan Inc., Pleasanton, CA, USA) using SerialEM acquisition software (Mastronarde, 2005). Tilt-series were recorded (at a nominal magnification of 29,000×) as described above for work with the F30. Tilt-series were aligned by a fiducial-less, patch-tracking alignment method available in the IMOD software package and built into tomograms. The tomograms shown here were processed using a Nonlinear Anisotropic Diffusion filter (Frangakis and Hegerl, 2001) to reduce image noise. To compensate for compression arising during sectioning, some images were stretched. The images were rotated in Adobe Photoshop (Adobe Systems Inc., San Jose, CA, USA) to orientate the knife marks parallel to the *Y*-axis. A distortion stretching the images along the cutting direction assuming an average 50% compression factor (Al-Amoudi et al., 2005; Pierson et al., 2011) was applied.

#### 2.1.3 Averaging of tomographic sub-volumes with PEET

**Supplementary Figure 1:**
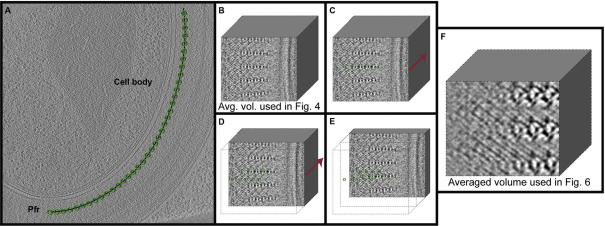
PEET averaging method used for the PFR. (A) Overview of the cell body and PFR, showing model points (green rings) placed along the PFR structure (following *x*, *y*-axis). Each of the model points represent the center of a sub-volume extracted, aligned and averaged using PEET. (B) represents the averaged volume obtained and used in Fig. 4. (C–E) shows how in order to improve the signal to noise ratio further, model points (green rings) were placed on the eye structures of the distal region of the PFR at different depths (red arrow) through the averaged volume. (Hence, the model points are placed this time along the *z*-axis). (F) The averaged volume obtained after running PEET a second time. This volume was used in Fig. 6.

To select sub-tomograms of the PFR for averaging, an open contour of 49 points, 56 nm apart was drawn through the region of interest. The tomogram subvolumes surrounding those 49 points were then aligned using PEET (Particle Estimation for Electron Tomography; http://bio3d.colorado.edu/PEET/): a software package that aligns similar structures by cross-correlation and averages sub-volumes of repetitive structural elements (Cope et al., 2010; Nicastro et al., 2006). An initial alignment was obtained using normalized cross-correlation on a 168^3^ pixel volume. For this, Euler search angles were initially set to 24° and refined during the search down to 1.5° after 4 iterations. The procedure was then reiterated on the resulting average, by selecting 26 new subvolumes along an axis perpendicular to those original 49 subvolumes, to produce a final average with improved signal to noise ratio (Supplementary Fig. 1). For parts of Fig. 5, a low-pass filter was applied to remove further noise.

## 3 Results

### 3.1 Structure of the flagellum in frozen hydrated sections

To investigate the *T. brucei* flagellar structure by cryo-EM and cryo-ET we subjected procyclic cells to high-pressure freezing and vitreous sectioning. In these sections, cross-sections of the flagellum appear round or elliptical (Fig. 1D–F). The PFR proximal and distal region shows repetitive slanted electron dense lines (Fig. 1D). Furthermore, the flagellar membrane is separated from the cellular membrane by a ∼20 nm extracellular space (numbers are not exact due to compression during sectioning). The rest of the area looks similar to what has been previously been described in chemically fixed sections (Sherwin and Gull, 1989). In the section shown in Fig. 1F the PFR appeared thinner than elsewhere along the flagellum length. Judging from the close proximity of the Golgi apparatus that is clearly visible right below, and the large distance between the sub-pellicular MTs in the FAZ region the section shown in panel 1F is probably close to the flagellar pocket where the PFR originates. This is further supported by the large distance between the sub-pellicular MTs in the FAZ region, this larger gap has been seen previously by plastic section tomography just outside the flagellar pocket (our unpublished data).

### 3.2 Extracellular ‘staples’ appear to attach the flagellum to the cell membrane

**Supplementary Figure 2:**
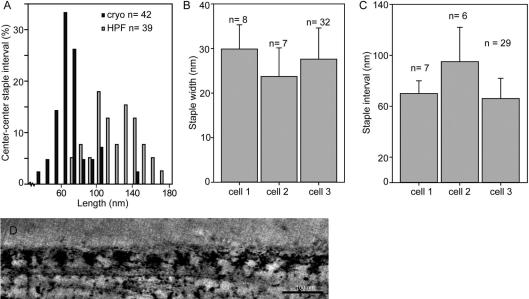
The staples are unlikely the same structure as maculae adherens. (A) The intervals measured from the center of one staple to the center of the next, from three different plunge frozen whole hydrated cells or five different high pressure frozen, freeze substituted and sectioned cells. (B–C) Comparison of staple width and interval length in the three different frozen hydrated cells show now clear difference between cells in these parameters. (D) A 10 nm thick tomographic slice of maculae adherens found in a cell that had been high pressure frozen, freeze substituted and sectioned.

**Fig.2:**
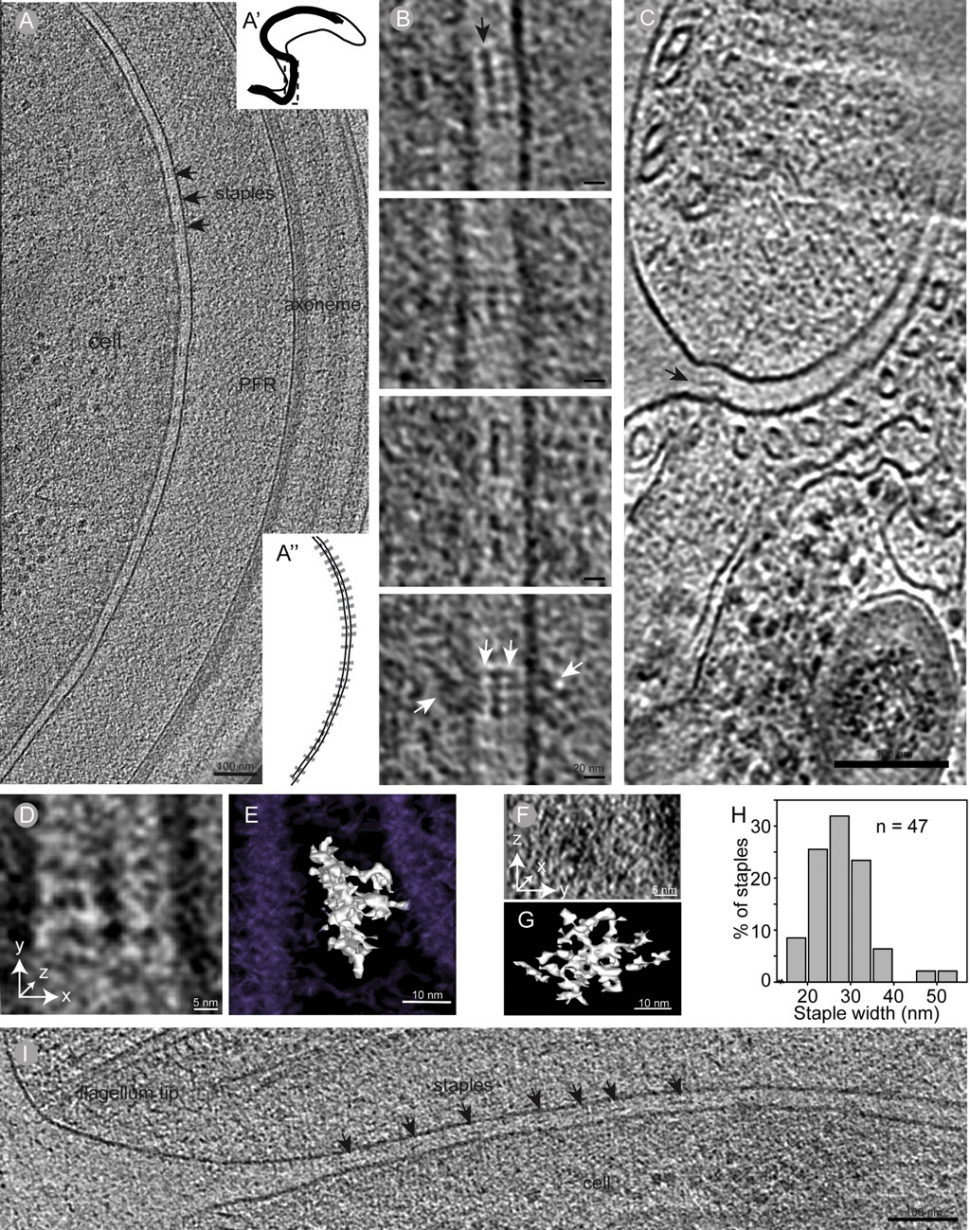
The flagellum is connected to the cell body with multi-domain protein assemblies that span both membranes. (A) A ∼30 nm thick tomographic slice of the region where the flagellar and cellular membranes show electron dense structures (arrows) spanning the space between them. Note the electron dense trapezoid shapes extending into the cytoplasm and flagellum on the sides of each electron dense extracellular structure, giving the connection a bowtie-like appearance. (A′ shows the region of the cell imaged in A). The insert A″ is a cartoon of the image shown in A, with the membranes and extracellular electron densities shown in black and the electron dense trapezoids shown in gray. (B) Higher magnification images showing the extra cellular structures, which consists of an electron dense midplate (arrow), with filamentous structures extending to both sides and into the cytoplasm/flagellum (white arrows). (C) An extra-cellular electron density (arrow) is also shown in this 20 nm thick slice of a tomogram acquired from a frozen hydrated section. Note that there is only one density visible in this cross-sectional view, indicating that only one row of these structures is present. (D and F) 7 nm thick tomographic slice of a sub-tomogram average of 30 staple structures. (E and G) The 3D isosurface of the membranes (purple) and the staple (white) of the subtomogram average. (H) The anterior end of the cell, with the staples (arrowheads) between flagellar and cell membranes.

Cryo-EM of plunge frozen whole cells enables detailed investigations of the molecular structures that attach the flagellum to the cell body, without the compression seen in frozen hydrated sections. In the areas where the flagellum was the closest to the cell body (membrane spacing = 26 ± 2 nm), we found electron dense extracellular structures situated along the flagellar axis at regular intervals (71 ± 20 nm center–center; *n* = 42; Fig. 2A and Supplementary Fig. 2). These structures appear to bridge the cellular and flagellar membranes and their width and spacing does not vary significantly between cells (Supplementary Fig. 2). On both sides of these structures, a diffuse, electron-dense trapezoid-shaped area extends into the cytoplasm as well as into the flagellar lumen (Fig. 2A–C; Supplementary Movie 1).

The “staples” define a distinct mid-plate parallel to the flagellar axis with fibrous densities on either side linking the cellular and flagellar membranes (Fig. 2B). Connecting fibrous material also appears to continue into the flagellum as well as the cytoplasm on either side of the staple. Views along the flagellar axis, obtained by cryo-sectioning, reveal a single row of these extracellular connections between the cell and the flagellum (Fig. 2C). This view also shows that the ‘staple’ is as deep as it is wide. Indeed, subtomogram averaging of 30 staple structures reveals its plate-like structure, where most of the fibrous connections to the membranes were lost, indicating their great structural flexibility (Fig. 2D–G). The staples themselves vary in width between 16–52 nm (average 27 ± 7 nm; Fig. 2H) and were found all the way to the anterior end of the cell with the same size and shape (Fig. 2I).

### 3.3 Vitrified sections of the flagellum transition zone and basal bodies

**Fig.3:**
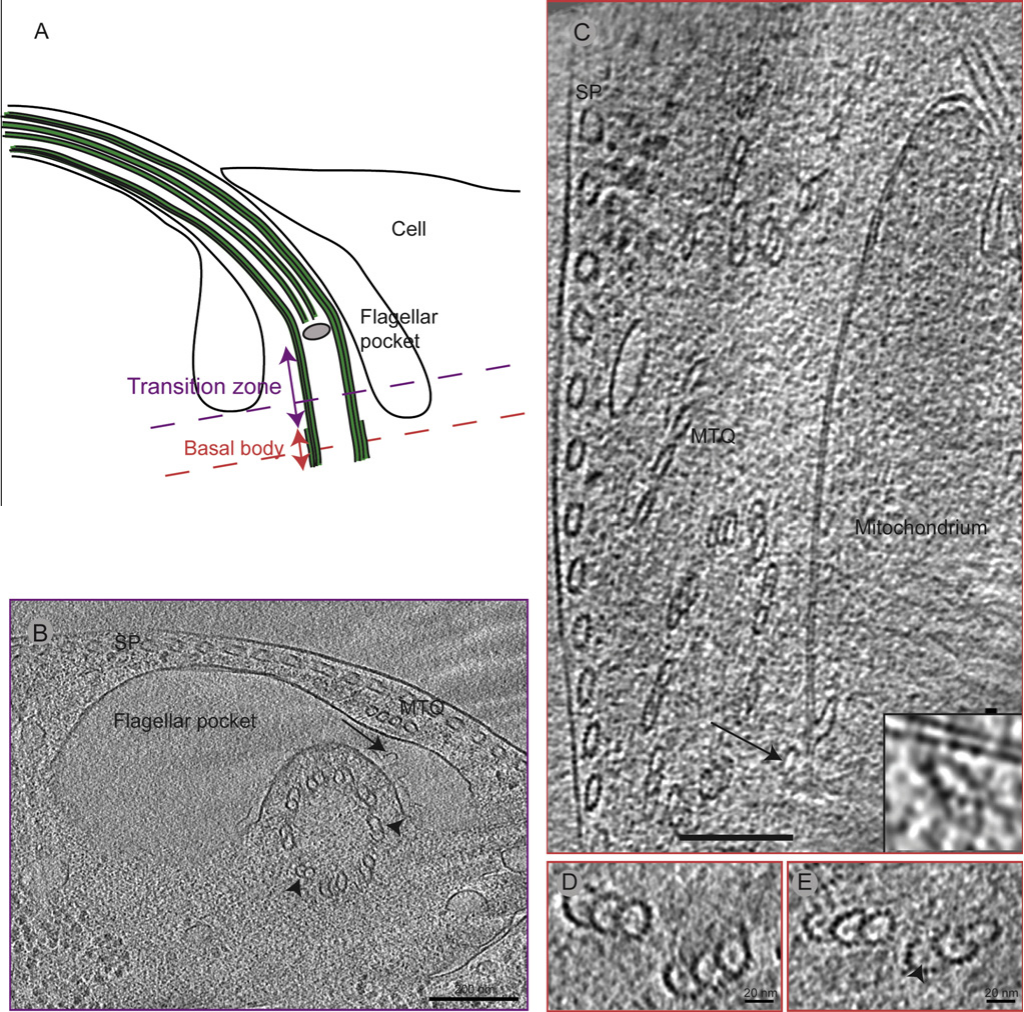
Structure of the intracellular part of the flagellum. (A) A cartoon showing the flagellum inside the flagellar pocket in longitudinal view. The purple dashed line in the transition zone shows the approximate location of the image shown in (B), the red line shows the location of C–E. (B) The transition zone of the flagellum, found inside the flagellar pocket, as seen in a 30 nm thick section of a vitreous section tomogram. Note the electron densities in the doublet microtubules (arrowheads). The collarette (arrow) forms a lobed line on the outside of the flagellum. The microtubule quartet (MTQ) wraps around the pocket, to join the sub-pellicular (SP) microtubules at the cell surface. (C) The basal bodies in a 50 nm thick slice of a vitreous section tomogram. A previously unidentified microtubule (arrow) is found close to the mitochondrial membrane. (D–E) Triplet microtubules from a second vitreous section tomogram containing basal bodies. The slices are 50 nm thick and displays electron densities inside A, B, and C tubules (arrowhead).

The flagellum originates beneath and passes through a large membrane invagination called the flagellar pocket (Fig. 3A). This intra-cellular region is found at the widest part of the cell, and thus can only be imaged by cryo-ET on vitrified sections; whole cells are too thick.

The flagellar pocket consists of smooth membranes, and along its edge the MTQ was clearly visible (Fig. 3B). Around the flagellum, the collarette structure (Lacomble et al., 2009; Vickerman, 1973) was partially visible (arrow in Fig. 3B) and the axonemal doublet microtubules in the proximal part of the transition zone of the flagellum were visible in cross-section. Remarkably, there are distinct electron densities in the center of most of these microtubules.

Below the flagellar pocket, the basal bodies can be found next to a mitochondrion (Fig. 3C). The basal body reveals the typical microtubule triplet structure commonly found in centrioles and basal bodies in a wide range of species (Allen, 1969; Gould, 1975; McKean et al., 2003; O’Toole et al., 2003; Pearson and Winey, 2009; Wolfe, 1970). Interestingly the triplet microtubules found here show distinct densities within their lumen (Fig. 3D–E); compare with (Garvalov et al., 2006; Nicastro et al., 2006; Sui and Downing, 2006).

Most remarkably, we discovered a previously undescribed microtubule located between the mitochondrion and the basal body (arrow and insert). This represents the first microtubule that has been found to float free in the cytoplasm of *T. brucei*, however, this could be the beginning of a new MTQ.

### 3.4 Detailed structural analysis of the PFR

**Fig.4:**
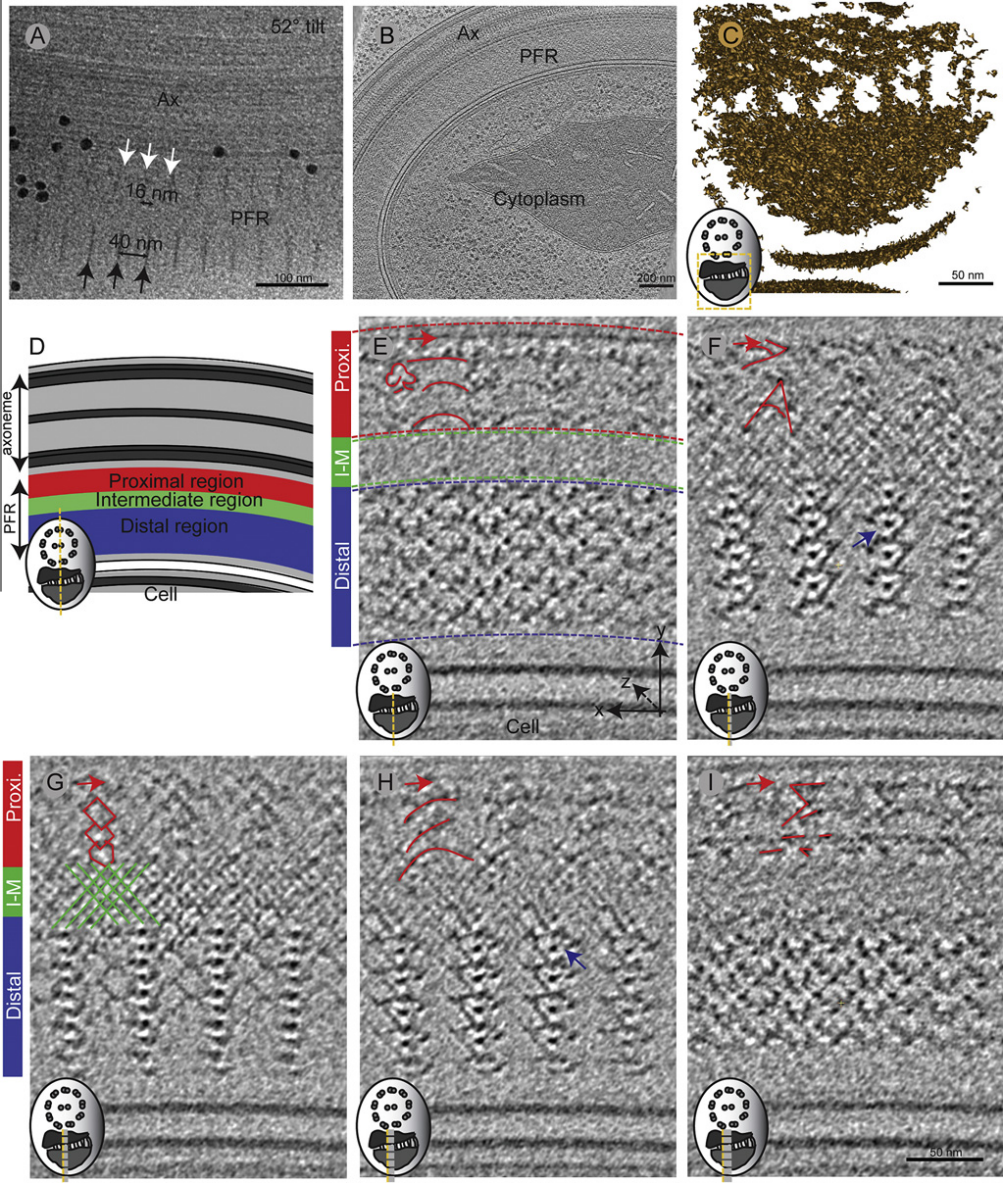
Structure of the para-flagellar rod (PFR). (A) A cryo-electron micrograph of a detergent extracted and plunge frozen cell, at 52° tilt, showing the PFR’s repetitive units in the proximal region (white arrows) and in the distal region (black arrows). The offset between the proximal and distal repeats are 16 nm (black double arrow). (B) A 30 nm thick tomographic slice of the intact flagellum, which was used for sub-tomogram averaging of the PFR structure shown in E–I. (C) Isosurface of the sub-tomogram average reveals a characteristic PFR structure. (D) Cartoon shows a trypanosome flagellum in longitudinal orientation, sectioned parallel to its axis. The microtubules in the axoneme (gray) lie parallel to the PFR, which is divided into axoneme-proximal (red), inter-mediate (green) and distal regions (blue). (E–I) 10 nm thick slices of a sub-tomogram averaged reconstruction, showing features within one repeat of the pattern formed by the electron densities. Their distribution in *z* as indicated by the yellow line in the cartoon: (E) 0 nm, (F) 14 nm, (G) 6 nm, (H) 6 nm and (I) 14 nm distance in *z* from the previous slice. The red and green outlines highlight the electron densities in the proximal and intermediate region, respectively, and the cartoon in the bottom left corner shows the present image’s locations (yellow line) to the previous images (gray lines).

The PFR is a paracrystalline structure; its repeats become particularly well visible in detergent-extracted plunge frozen cells at 52° tilt (Fig. 4A). The removal of membranes provided extra contrast that revealed the repetitive units in the regions both proximal and distal to the axoneme. In this tilted view, these repetitive units were 40 ± 1 nm long (*n* = 12) and at a 16 ± 2 nm (*n* = 10) offset to each other, corresponding to a 51 ± 1 nm repeat with a 20 ± 3 nm offset at 0°. To improve the signal to noise ratio in our images, we averaged sub-tomograms using PEET (Cope et al., 2010; Nicastro et al., 2006). These volumes were extracted from a tomogram of a slightly bent flagellum on a whole-mount plunge frozen cell (with unperturbed membranes; Fig. 4B; Supplementary Movie 2). 3D rendering of the averaged volume by density thresholding yielded a model that resembles previous images of the PFR (Fig. 4C; Supplementary Movie 3) (Gadelha et al., 2006; Portman et al., 2009; Vickerman, 1962), confirming the successful averaging.

The 3D map obtained from averaged volumes revealed many novel features of the PFR. Fig. 4D illustrates the different regions of the PFR in longitudinal view. For an optimal viewing of the repetitive elements we projected 10 nm thick tomographic slices (Fig. 4E–I; Supplementary Movie 4), but at different *z*-heights (as represented in by the yellow line in the cross-section cartoon).

Within one repeat, the proximal region features a three-layered structure of arches interrupted by cloverleaf-like electron densities (red outline; Fig. 4E). The assembly shown in Fig. 4E comes from 14 nm further down along the *z*-axis. There, the arches are replaced by A-shaped densities (Fig. 4F), which change into a pattern that appears like columns of stacked diamonds another 6 nm further down (Fig. 4G). Yet another 6 nm further down, we found electron dense features resembling stacked arches (Fig. 4H). Another 14 nm deeper, the electron densities show sharper edges (Fig. 4I). Throughout, the densities in the proximal region appeared connected into a line at the side closest to the axoneme (red arrow).

The intermediate region (green bar; Fig. 4E) displayed less pronounced features, but it showed a fine mesh of lines that overlaid each other at a ∼90-degree angle (green outline; Fig. 4E–I).

The distal region (blue bar; Fig. 4E) appears to be the most structured region of the PFR. Note that in Fig. 4F the electron density has a less dense side towards the left (blue arrow), but in 4H this less electron dense side is found to the right (blue arrow), making the electron densities appear like mirror images of each other.

### 3.5 The proximal and intermediate region is repetitive along the axis of the flagellum

**Fig.5:**
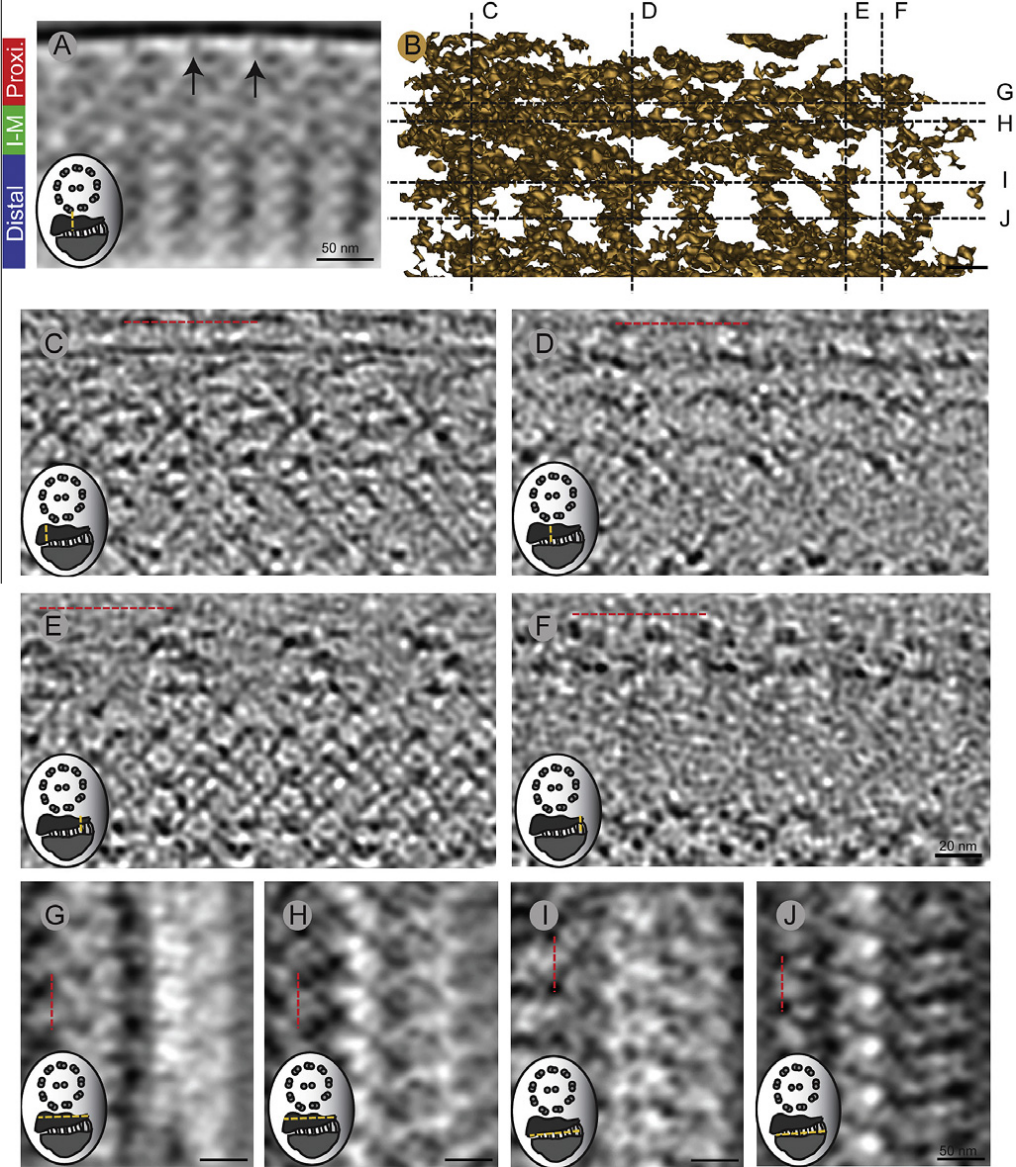
The proximal and intermediate regions of the PFR. (A) Low-pass filtering of the averaged volume reveals clear connections from the proximal region to the microtubule doublet the closest to the PFR. (B) An isosurface of the unfiltered volume reveals structural features of the proximal region and its connection to the intermediate region. Dotted lines show where in the PFR the tomographic slices of C–J were extracted. (C–J) Shows 10 nm thick slices of the averaged tomogram. (G–J) has been low-pass filtered to reveal the repeat (highlighted in a dotted red line). Yellow lines show the orientation of the slice shown.

After a low-pass filtering, clear connections between the proximal region and the closest microtubule doublet are revealed (arrows; Fig. 5A). An isosurface of the unfiltered volume is noisy, yet reveals important structural details of the proximal and intermediate region (Fig. 5B). In this cross-sectional view, the proximal region has one thicker side (left) and one thinner side (right). In the thick area, parallel electron densities appear anchored at three of the seven stalks found in the intermediate region (the stalks connect the proximal with the distal region). Above the central stalk, there is a void, causing a clear break in the symmetry along this axis. On the thinner side of the proximal region, the electron densities do not form clear parallel lines. The interface between the proximal and intermediate region is slightly ‘V’ shaped with the bottom of the ‘V’ off centered towards the thicker side.

In Fig. 4, we showed the ultra-structure inside one of the repeated units. Here, we show the structure of the proximal and intermediate regions at selected spots throughout the PFR (Fig. 5C–J). Inside the PFR, the proximal and intermediate regions show clear repeated structure along the length of the flagellum (Fig. 5C–J). The cross-patterned electron densities in Fig. 5E are what form the stalks that connect the proximal and distal PFR region.

We conclude that the proximal and intermediate regions are not symmetrical in flagellar cross-section, but highly structured and repetitive along the length of the flagellum, and therefore quite different to the distal region that is repetitive in 3D.

### 3.6 The distal PFR region consists of highly ordered arrangement with orthorhombic crystal packing

**Fig.6:**
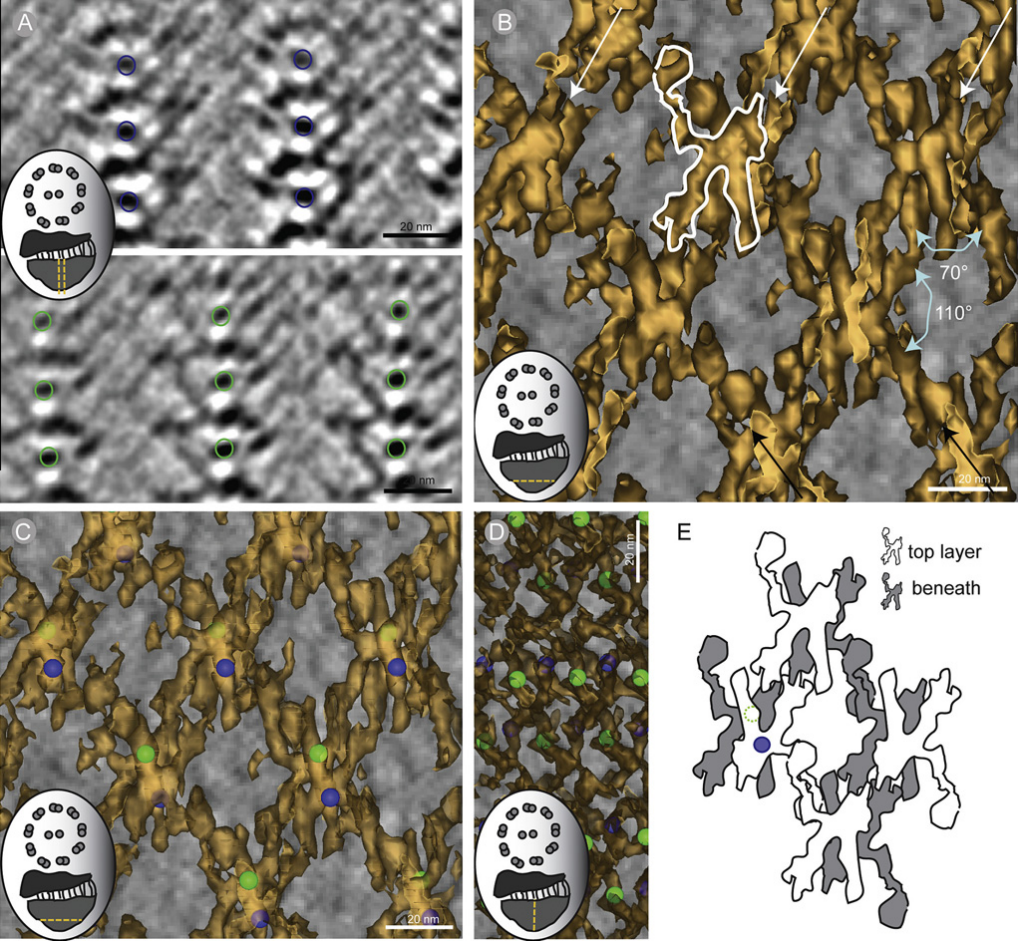
The repetitive unit in the distal region of the PFR is packed as an orthorhombic crystal. (A) To learn how the repetitive units were oriented relative to one another we put a green or blue sphere (shown here as rings) at a recognizable location on two 10 nm thick slices of the final-average. Green rings were placed 12 nm above in *z*, marking objects similar to those marked with blue rings. (B) Isosurface generated by density thresholding of the PEET average reveals two sets of parallel lines (white and black arrows) that intersect at an angle of 110°, generating a cross pattern. By closer examination, a repetitive unit shaped like a man holding a racket (outlined in white) was found in the density map. These repetitive units appeared to be oriented in a feet-to-feet and head-to-head fashion. (C) When seen inside the iso-surface, the green and blue spheres were located central in the repetitive unit, and clearly revealed that in the *z*-axis the layers of repetitive unit are lying head to foot. (D) The iso-surface model has been rotated 90° around the *Y*-axis to show the same orientation as in B). (F) A cartoon showing how the repetitive unit creates the pattern seen in the distal region of the PFR. The blue circle represents the location of the blue sphere in (D) and the dotted green ring the green sphere on the layer behind.

We pursued a more detailed map of the repetitive unit in the distal region of the PFR by creating a final average of the averaged volume. The final averaged 3D map (Fig. 6A) shows the structure of the PFR distal region with improved signal to noise ratio (compare with Fig. 4F and H). We used electron density thresholding to render a 3D surface view of the averaged 3D map. This model reveals two sets of parallel lines that meet at an angle of 110° (Fig. 6B), suggesting an orthorhombic unit cell. A minimal repetitive unit can be identified in this 3D model (Fig. 6B; white outline); these units are aligned head-to tail in the *x*–*y* planes of the tomogram.

To understand how this minimal unit forms the repetitive pattern that makes up the PFR’s distal region along the *z*-axis, we identified easily recognizable features in the final average (blue and green rings; Fig. 6A).These have been marked with a sphere that shows up in the 3D model (Fig. 6C–D). The distribution of the spheres shows that the minimal units are stacked in *z*-direction with one unit on top of its mirror image (Fig. 6D). Thus, the PFR distal region is built as an orthorhombic crystalline arrangement.

## 4 Discussion

The study presented here constitutes the first cryo-ET structural investigation into the unperturbed eukaryotic flagellum of *T. brucei*, still attached to the cell body and with all membrane systems intact. Except for some work on sea-urchin sperm flagella (Nicastro et al., 2005, 2006), previous cryo-EM studies of flagella have been performed on isolated flagella where the membranes have been removed by detergent extraction (Bui et al., 2008, 2009; Heuser et al., 2009; Koyfman et al., 2011; Movassagh et al., 2010; Nicastro et al., 2006; Pigino et al., 2011; Sui and Downing, 2006; Takazaki et al., 2010), The same is true for a more recent study on the *T. brucei* PFR (Koyfman et al., 2011). Although frozen-hydrated specimens and vitrified sections thereof are still not free of artifacts (e.g. compression, distortions (Bouchet-Marquis and Hoenger, 2011)) vitrification and vitrified sectioning is currently accepted as the best possible preservation method for molecular studies of cell organelles and supramolecular assemblies (down to 2–3 nm resolution) in relatively large biological systems (Leis et al., 2009; McIntosh et al., 2005).

Our preparations with intact membranes revealed a novel extracellular component in the flagellar attachment zone, the staple-like structures described in Fig. 2. We suggest these structures attach the flagellum to the cell body, since they are located exactly where such a connection must reside: where a constant distance between the two membranes is found. In this region, maculae adherens have been shown in bloodstream form of *T. brucei* (Vickerman, 1969) and procyclic cells (Sherwin and Gull, 1989). However, in bloodstream cells, these structures were found 95 nm apart (Vickerman, 1969), which differs from the distance measured between ‘staples’ in this paper. This could be due to differences in organization of this region between life cycle stages, so we measured the distance center–center between maculae that are easily recognized in high pressure frozen and freeze substituted procyclic cells (122 ± 25 nm; *n* = 39; Supplementary Fig. 2). Therefore, we conclude that the staples are a new feature of the FAZ, and unlikely a component of the previously described maculae adherens as they are found more frequently along the flagellar axis.

Even though these membrane spanning protein complexes with extracellular domains are smaller than other cell-to-cell adheres complexes such as desmosomes and tight junctions, the electron dense center in the extracellular domain of the staple is similar to the electron dense region where the cadherins are found in desmosomes (Al-Amoudi et al., 2011). However, there are no cadherins, or indeed other intermediate filaments, found in the *T. brucei* genome (Berriman et al., 2005), indicating that even if some structural similarities are seen, the components are different. Although the apparent complexity of the staple structure and its size suggests that this may well be a multi-protein complex, RNAi of a single protein, Fla1 (LaCount et al., 2002), will release the flagellum from the cell body in *T. brucei*, interfering with cytokinesis and decreasing viability. Likewise, RNAi of a FAZ-filament component, FAZ1, decreases cellular viability (Vaughan et al., 2008), revealing the importance of this entire region.

The general shape of a frozen hydrated flagellum differs substantially from the ones prepared by chemical fixation. After chemical fixation, the flagellar and cellular membranes are wrinkled, and there is often a waist-like indentation that forms below the axoneme and above the PFR (Gadelha et al., 2006; Portman et al., 2009; Rocha et al., 2010). Frozen hydrated samples reveal membranes that are smooth, and in cross-section the flagellum appears perfectly oval or circular. The vitrified sample reveals an additional volume in the flagellum. This space is near doublets 3–4 and 7–8 where intraflagellar transport occurs (Absalon et al., 2008).

Finally, our cryo-ET approach has revealed detailed 3D architecture of the PFR, a structure whose contribution to kinetoplastid motility is not fully understood. Previously, it has been suggested that the role of the PFR is to act as an internal elastics bending resistance, based on the impaired swimming capabilities found in RNAi mutants of the PFR2 protein in *Leishmania mexicana* (Santrich et al., 1997). In *T. cruzi*, (the causative agent of Chagas disease) atomic force microscopy and transmission electron microscopy of chemically fixed, quick frozen, freeze fractured replicas revealed that the periodicity of the crossing filaments in the PFR changes depending on whether the flagellum is bent or straight (Rocha et al., 2010) indicating intrinsic flexibility in that system. Recently, this was also found to be the case in *T. brucei* where different flagellar bending states were examined by cryo-ET. There the authors suggested that the PFR may act as a biological jackscrew, a force amplifying tool (Koyfman et al., 2011). Our data (presented in Figs. 4 and 6) originate from a bent flagellum, with angles that coordinate well with the angles revealed in that paper attributed to an outward bent flagellum. However, as there is no electron density forming a bridge between the two sides of the ‘jackscrew’, such as the screw used in a car jack, we do not see how this structure could amplify force. Therefore, it is still unclear whether a specific, merely structural role of the PFR in flagellum motility can be added to the role already established in compartmentalization of biochemical processes within the flagellum. In this paper, we have further revealed the extreme complexity of the PFR structure, which provides a map into which biochemical compartments can be identified to further understand the role of the PFR in the kinetoplastids.

## 5 Conclusions

Using cryo-ET we revealed a novel structure that we assume is involved in the attachment of the flagellum to the cell in *T. brucei*. Furthermore, we have identified the macromolecular organization of the paraflagellar rod, whose distal region consists of an orthorhombic crystal.

## Appendix A Supplementary data

Supplementary video 1 Staples present in one line between the cell body (left) and the flagellum (right). The movie shows tomographic slices of a plunge frozen whole cell. Each image shows a 15 nm thick slice, moving through the volume in increments of 3 nm. Scale bar 100.

Supplementary video 2 The ultrastructure of the PFR, seen lying lengthwise to the left with the cell membrane to the right. The volume moves through the whole width of the PFR, where each image is a 1 nm thick tomographic slice of the subtomogram average that has been filtered using NAD. Scale bar 50 nm.

Supplementary video 3 Isosurface of the PFR. The movie starts with a cross sectional view of the PFR, and then rotates to reveal the repeats of the three regions. The isosurface was made using the non-filtered averaged subtomogram. Scale bar 50 nm.

Supplementary video 4 Isosurface containing only one PFR repeat. The raw data is as Suppl. Movie 3, but here only one repeat of the PFR has been extracted. Scale bar 50 nm.
