# Supplementary figures and images for: Cryo-electron tomography and 3-D analysis of the intact flagellum in Trypanosoma brucei
Source: J Struct Biol. 2012 May;178(2):189–98. doi: 10.1016/j.jsb.2012.01.009 (PMC3355306; doi:10.1016/j.jsb.2012.01.009)

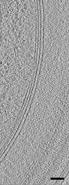

Supplement: Supplementary video 1 — Staples present in one line between the cell body (left) and the flagellum (right). The movie shows tomographic slices of a plunge frozen whole cell. Each image shows a 15 nm thick slice, moving through the volume in increments of 3 nm. Scale bar 100. [file mmc1.jpg]

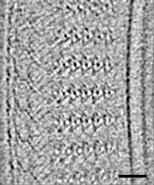

Supplement: Supplementary video 2 — The ultrastructure of the PFR, seen lying lengthwise to the left with the cell membrane to the right. The volume moves through the whole width of the PFR, where each image is a 1 nm thick tomographic slice of the subtomogram average that has been filtered using NAD. Scale bar 50 nm. [file mmc2.jpg]

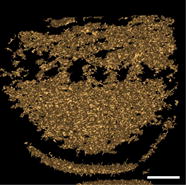

Supplement: Supplementary video 3 — Isosurface of the PFR. The movie starts with a cross sectional view of the PFR, and then rotates to reveal the repeats of the three regions. The isosurface was made using the non-filtered averaged subtomogram. Scale bar 50 nm. [file mmc3.jpg]

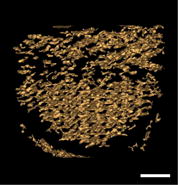

Supplement: Supplementary video 4 — Isosurface containing only one PFR repeat. The raw data is as Suppl. Movie 3, but here only one repeat of the PFR has been extracted. Scale bar 50 nm. [file mmc4.jpg]
